# The Gordon Research Seminar & Conference on Parkinson’s disease: state of the Science 200 years after James Parkinson’s essay on the Shaking Palsy

**DOI:** 10.1038/s41531-017-0028-y

**Published:** 2017-08-09

**Authors:** J. T. Greenamyre, B. R. De Miranda, M. L. Bucher, A. B. Singleton, M. G. Tansey

**Affiliations:** 1 Pittsburgh Institute for Neurodegenerative Diseases, University of Pittsburgh, Pittsburgh, PA USA; 2 Laboratory of Neurogenetics, National Institute on Aging, Bethesda, MD USA; 3 Department of Physiology, Emory University School of Medicine, Atlanta, GA USA

## Abstract

The first-ever Gordon Research Seminar on Parkinson’s disease was held in conjunction with the second-ever Gordon Research Conference on Parkinson’s disease at the Grand Summit Hotel at Sunday River in Newry, Maine, from June 24–30. The Gordon Research Seminar brought together graduate students and postdoctoral researchers to network, learn first-hand about life with Parkinson’s disease, listen to and present Parkinson’s disease science, and hear about a variety of relevant career options. The Gordon Research Conference began as the Gordon Research Seminar concluded and was attended by a broad, international mix of junior and senior scientists from academia and industry. It was organized into eight outstanding scientific sessions in which cutting edge science, much of it unpublished, was presented. Among attendees, there was universal praise for the content and organization of the meeting, and for its open and welcoming ambiance.

In this, the 200th anniversary of James Parkinson’s Essay on the Shaking Palsy, the first-ever Gordon Research Seminar (GRS) on Parkinson’s disease (PD) was held at the Grand Summit Hotel at Sunday River in Newry, Maine, on June 24th and 25th. The GRS, organized by and for postdoctoral researchers and graduate students, was chaired by Briana De Miranda and co-chaired by Meghan Bucher. The GRS was followed immediately by the second-ever Gordon Research Conference (GRC) on PD (June 25–30), which was organized by Andy Singleton (chair) and Tim Greenamyre (vice-chair). Both meetings were over-subscribed, and a total of 200 participants from at least 15 different countries (representing four continents) made the long trek to Sunday River (Fig. 1). The relative isolation of this excellent venue enhanced the chances for interactions and the planning of new collaborations. There was a nice mix of trainees, and junior and senior scientists, and there was broad participation by academic and industry scientists, program staff from the National Institutes of Health (NINDS and NIEHS) and representatives of foundations (the Michael J Fox Foundation and the Parkinson’s Foundation). Also in attendance for the entire GRS/GRC was an emeritus professor of chemistry and former GRC board member who was recently diagnosed with PD. His active participation and interactions with PD researchers gave important context to the scientific presentations.

**Fig. 1:**
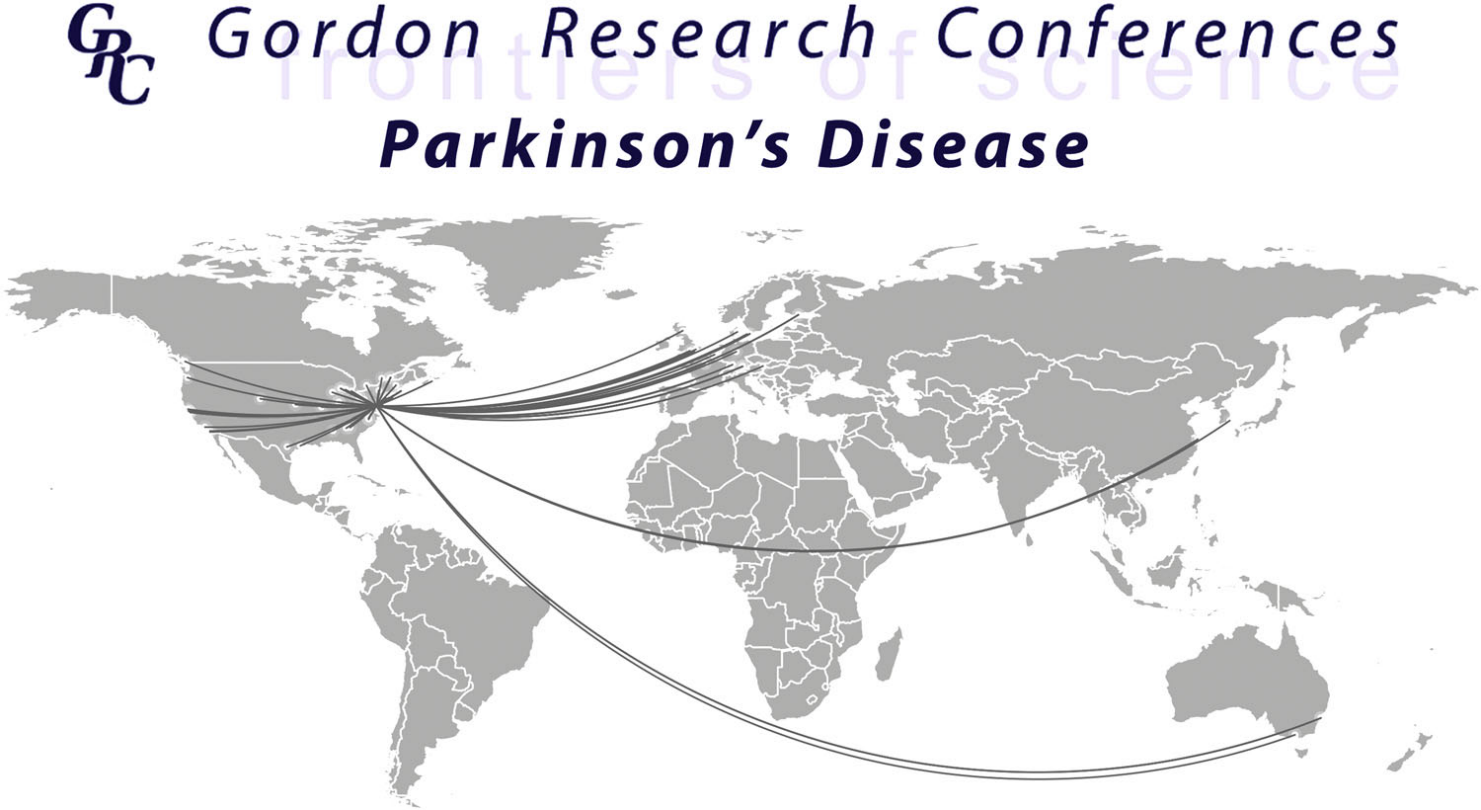
The Gordon Research Seminar & Conference on Parkinson’s disease was attended by 200 scientists from 15 nations representing four continents. There were almost 50 invited speakers, and more than 100 posters were presented. Three Carl Storm Underrepresented Minority Fellowships were awarded by the GRC

The week of Parkinson’s science began with the GRS—and an outstanding keynote presentation by Lorenz Studer on “Modeling and Treating Parkinson’s Disease Using Human Pluripotent Stem Cells”. Then, in addition to poster sessions and a series of 20-min talks by selected trainees, there was a moving and articulate first-hand discussion of what it’s like to have PD by Nicole L, a young-onset PD patient, who was diagnosed at the age of 28 after giving birth to her first daughter. For some of those in attendance, this was their first opportunity to interact directly with someone with the disease. There was also a discussion of issues related to publishing in a high-impact journal by Orla Smith (*Science Translational Medicine*). The meeting ended with a career development panel in which representatives from industry, a PD foundation, the NIH, professional publishing, and academics discussed their career trajectories and perspectives on job opportunities in their respective fields. There was unanimous praise for the organization and content of the GRS, and participants spoke highly of every aspect of the meeting, especially the opportunity to network with new colleagues, juniors, and seniors alike. Importantly, the GRS served as an ‘ice-breaker’ for the trainees, after which they no longer felt intimidated or reluctant to ask questions at the GRC or to approach senior scientists. At its conclusion, Soumitra Ghosh and Collin Bantle were elected chair and vice-chair of the 2019 GRS.

Where the GRS left off, the GRC began (a few hours later). As discussion leader for the keynote session (“Informing Etiology Through the Clinic”), Warren Olanow brought home the practical difficulties inherent in designing, implementing, and interpreting a trial of an agent designed to be “disease-modifying” or “neuroprotective” in PD. In this time of optimism for a breakthrough in the treatment of PD, many in the audience commented that this was a sobering dose of reality. After this introduction, Bill Langston then delivered the keynote address: “What Is Parkinson’s Disease and Why Is It Important for Understanding Biology”. The content was informative, provocative, controversial, and highly entertaining as it laid out the difficulties of defining “Parkinson’s disease” based on clinical, pathological, or genetic grounds. Echoes of his presentation spontaneously reverberated in subsequent talks and audience questions for the remainder of the meeting.

As with the first GRC in 2015, the 2017 meeting was not comprehensive; it was only able to address a limited (but different) set of topics of importance in PD. Like all GRCs, there were eight scientific sessions: (1) Parkinson’s Disease Genetics and Systems Biology; (2) Gene-Environment Interaction; (3) LRRK2: From Protein, Through Cell Biology to Therapeutic Intervention; (4) The Biology of Aging and Parkinson’s Disease; (5) Protein Handling and Trafficking; (6) Parkinson’s Disease Models: Improving Predictive Value; (7) SNCA: From Protein, Through Cell Biology to Therapeutic Intervention; and (8) Inflammation and Immunity in Parkinson’s Disease. A couple of the topics, those on LRRK2 and α-synuclein (SNCA), are sufficiently mature that they were each organized into ‘vertical’ sessions spanning from basic biology to ongoing therapeutic development programs by Pharma. Comparisons by industry scientists of the PD therapeutic development programs (for LRRK2 and α-synuclein) to those in Alzheimer’s disease (for beta-amyloid), which are farther along, provided important context—and they suggest that the PD community needs to be prepared for the long game, while simultaneously working to accelerate progress. This also highlighted the need of GRS/GRC meetings to stimulate conversation, potentiate collaboration, and grow the next leaders in the field.

One aspect of the program that was enjoyable for both speakers and audience members alike was the inclusion of experts from outside the PD field, and those whose research findings have led them into PD. Thus, Michel Desjardins described the novel phenomenon of mitochondrial antigen presentation, which potentially ties together mitochondria (including parkin and PINK1), immunology, vesicle cycling, LRRK2, and α-synuclein (among others!). Dario Alessi, an expert in protein phosphorylation and signaling, discussed his groundbreaking work on LRRK2 and its physiology. Roberta Brinton led a session on aging in relation to PD, and Andrew Yoo discussed microRNA-based reprogramming of cells into neurons in that context. Similarly, Rick Morimoto discussed proteostatic mechanisms and defects in aging. In addition, Victoria Bolotina, an expert in calcium signaling and ion channels, described how she got into the PD field via her studies of PLA2G6 (PARK14) and its role in calcium homeostasis.

At the GRS and GRC, there was a great deal of participation—both formal and informal—by scientists from industry. Given that we are likely on the cusp of therapeutic breakthroughs in PD, this was essential. It is important to note that industry participation not only did not detract from the GRC policy of presenting unpublished data, it actually enhanced it. Many of the attendees commented that the combined GRS/GRC was one of the most open and welcoming conferences they had ever attended; all looked forward to the next GRS/GRC planned for 2019.

Not all the valuable activities at the GRC were strictly related to science. Roberta Brinton and Marie-Francoise Chesselet led the Power Hour, “designed to help address the challenges women face in science and support the professional growth of women in our communities by providing an open forum for discussion and mentoring.” Representatives from federal (NINDS & NIEHS) and foundation (Michael J Fox Foundation & Parkinson’s Foundation) granting agencies generously volunteered for a breakout session on funding opportunities for students and postdocs. Similarly, a breakout session was organized for trainees by industry attendees to provide information and answer questions about careers in the pharma/biotechnology sector. All these sessions were timely and well-received.

Toward the end of the GRC, at the business meeting, Malú Tansey was elected as vice-chair of the 2019 GRC to assist Tim Greenamyre in organization of the meeting; she will chair the 2021 conference. It is important to note, however, that a GRS/GRC on Parkinson’s disease in 2019 and beyond has not yet been granted by the Gordon Research Conferences. Nevertheless, given the popularity of the current meeting (the organizers had to turn away applicants when the GRS and GRC limits were reached), it is hard to imagine otherwise. The time is ripe; we are on the verge of amazing progress in PD, and we are growing the talent to make this happen. As James Parkinson said in 1817, “… there appears to be sufficient reason for hoping that some remedial process may ere long be discovered, by which, at least, the progress of the disease may be stopped.”

